# Characterization of Electrosynthesized Conjugated Polymer-Carbon Nanotube Composite: Optical Nonlinearity and Electrical Property

**DOI:** 10.3390/ijms13010918

**Published:** 2012-01-16

**Authors:** Afarin Bahrami, Zainal Abidin Talib, Esmaeil Shahriari, Wan Mahmood Mat Yunus, Anuar Kasim, Kasra Behzad

**Affiliations:** 1 Department of Physics, Universiti Putra Malaysia, UPM, Serdang, Selangor, 43400, Malaysia; E-Mails: afarin.bah@gmail.com (A.B.); esmaeil.phy@gmail.com (E.S.); mahmood@science.upm.edu.my (W.M.M.Y.); kasra.behzad@gmail.com (K.B.); 2 Science Faculty, Islamic Azad University, Eslamshahr Branch, 3314767653, Iran; 3 Department of Chemistry, Universiti Putra Malaysia, UPM, Serdang, Selangor, 43400, Malaysia; E-Mail: anuar@science.upm.edu.my

**Keywords:** conducting polymers, carbon nanotubes, optical properties, electrical characterization

## Abstract

The effects of multi-walled carbon nanotube (MWNT) concentration on the structural, optical and electrical properties of conjugated polymer-carbon nanotube composite are discussed. Multi-walled carbon nanotube-polypyrrole nanocomposites were synthesized by electrochemical polymerization of monomers in the presence of different amounts of MWNTs using sodium dodecylbenzensulfonate (SDBS) as surfactant at room temperature and normal pressure. Field emission scanning electron microscopy (FESEM) indicates that the polymer is wrapped around the nanotubes. Measurement of the nonlinear refractive indices (n_2_) and the nonlinear absorption (β) of the samples with different MWNT concentrations measurements were performed by a single Z-scan method using continuous wave (CW) laser beam excitation wavelength of λ = 532 nm. The results show that both nonlinear optical parameters increased with increasing the concentration of MWNTs. The third order nonlinear susceptibilities were also calculated and found to follow the same trend as n_2_ and β. In addition, the conductivity of the composite film was found to increase rapidly with the increase in the MWNT concentration.

## 1. Introduction

Conducting polymers are suitable alternative to replace metals in industry as they have the ability to withstand high electric fields with good environment stability and low cost of fabrication. Its physical properties are also not satisfactory for practical applications. This led to intensive research to achieve both high electrical conductivity and desirable optical and mechanical properties.

Polypyrrole (PPy) is a well-known conducting polymer, that has been studied by many researchers [1,2]. It is most frequently used in various device applications, such as in sensors [3,4] microelectronic devices [5] and composite materials [6,7]. Following the first report of the synthesizing of carbon nanotubes polymer nanocomposite, [8] many attempts have been made for combination of carbon nanotubes and polymers to produce materials with superior properties [9,10]. Carbon nanotubes (CNTs) have unique structural, electrical and optical properties. They are empty cylinders with diameters less than 100 nm and length on the micrometer scale and one of their features is their high surface to volume ratio. There are two different main methods for polymerization that is chemical and electrochemical method. However, this work focuses on the combination of the properties of multi-walled carbon nanotube (MWNT) and polypyrrole following an electrochemical method to the formation of the nanocomposite.

Both conducting polymers and CNTs have conjugated π bonds structure. The delocalized π electrons in CNT and PPy can bond together in nanocomposite to reduce energy of the system to form the PPy/CNT nanocomposite [11–13] and conducting polymer MWNT nanocomposites with core-shell structure are obtained [14]. MWNTs act as a dopant in the nanocomposite deposited on the working electrode. We use MWNTs as a conductive nanomaterial inside the PPy conducting polymer for developing the physical properties due to interfacial interactions between MWNT and the conducting polymer [15–17].

In the present work, we have investigated the structural, optical and electrical properties of the conducting polymer, polypyrrole and MWNT composite films prepared using electrodeposition.

Optical characterization of the nanocomposite samples was performed using a Z-scan method. Z-scan is a well-established method for the determination of nonlinear refraction and absorption and has been widely used in material characterization because it provides not only the magnitudes of real and imaginary parts of nonlinear susceptibility, but also the sign of the real part [18,19].

A large number of papers have been published on the synthesis and characterization of CNT and PPy nanocomposite films [20,21] but to our knowledge there are a few reports on the nonlinear optical properties of the PPy/CNT nanocomposite.

## 2. Experimental Details

In this study, the pyrrole monomer (Fluka) was distilled prior to use. Multiwalled carbon nanotubes (Nanostructure & Amorphous Materials) and sodium dodecylbenzensulfonate (Aldrich) were of analytical grade and used without further purification.

For the preparation for electrochemical polymerization, the ITO glasses were completely washed using sonicator bath. After dissolving the sodium dodecylbenzensulfonate (SDBS) in distilled water, the MWNTs with different weight ratio were dispersed in SDBS solution and sonicated for 4 hours. The ratio of nanotubes to surfactant was 1:10. Then pyrrole was dissolved in this MWNT/SDBS solution and again ultrasonicated for another 10 min, the monomer concentration was 0.l M Subsequently, the PPy/MWNT premixed solution was electropolymerized at +0.7 V for 5 min, in a three electrode electrochemical cell in which the ITO was used as a working electrode while a graphite rod and a saturated calomel electrode were used as the counter and reference electrode, respectively. The electrochemical polymerization was performed using a potentiostat (PS 605, USA) at room temperature. The current density was 0.5 mA/cm^2^. No other salts and solvents were added to the solution. The deposited polymerized PPy/MWNT thin films were washed with water and methanol to remove the electrolyte solution and dried under vacuum at room temperature for 24 hours.

The thickness of the samples was measured using a high surface profilermeter (AMBIOS TECHNOLOGY XP-200) which has an accuracy of ±10 nm. The linear refractive index and linear transmission coefficient were measured using an ellipsometer and fiber optics spectrophotometer (OCEAN OPTICS USB4000-FL), respectively. The electrical conductivity of the nanocomposite films samples was measured by a four point probe instrument. For nonlinear properties measurements, a single beam Z-scan method with closed and open aperture arrangements was used to measure the nonlinear refractive and nonlinear absorption coefficients. The measurements were carried out at room temperature using a CW beam diode laser operated at 532 nm wavelength (Coherent Compass SDL-532-150T). The beam was focused to a small spot using a lens and the sample was moved along the z-axis by a motorized translational stage. The power output of the laser beam measured at the focused point was 35 mW. The transmitted light in the far field passed through the aperture and the beam intensity was recorded by a photodiode detector. The laser beam waist ω_0_ at the focus length was 24 μm and the Rayleigh length was found to satisfy the basic criteria of a Z-scan experiment.

## 3. Results and Discussion

### 3.1. Morphology

**Figure 1:**
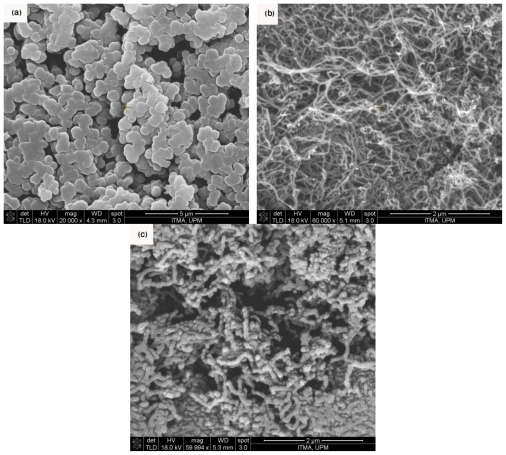
Field emission scanning electron microscopy (FESEM) image of (**a**) the Polypyrrole (PPy) formed by electrochemical polymerization without multi-walled carbon nanotube (MWNT); (**b**) the purified MWNTs; (**c**) the PPy/MWNT nanocomposite formed by electrochemical polymerization.

Figures 1a to 1c show FESEM (FEI Nova NanoSEM 230) images of the purified MWNTs, the PPy formed by electrochemical polymerization without MWNT, and the PPy/MWNT nanocomposite formed by electropolymerization. These figures show that the diameter of CNTs has been increased by electrochemical polymerization of pyrrole monomer onto the CNT as expected, and confirms the presence of CNTs inside the films. In addition, it is obvious that coated CNTs inside the film did not have any particular direction, and they are connected to each other by means of polymer.

The thickness of polypyrrole coated over the MWNT was in the range of 150**–**350 nm, and it was possible to control the thickness of the film by changing the experiment parameters, e.g., the time of polymerization or concentration of materials. From Figure 1 it is obvious the PPy formed under similar condition shows typical cauliflower morphology, with no nanocable-like morphology related to PPy/MWNT composite.

**Figure 2:**
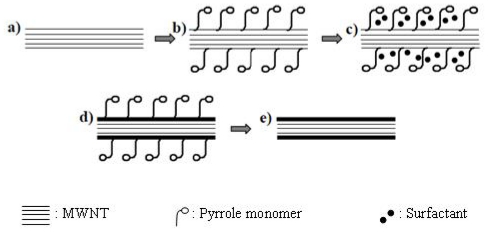
Schematic representation of the formation of the PPy/MWNT nanocomposite in the presence of sodium dodecylbenzensulfonate (SDBS) surfactant. (**a**) MWNT; (**b**) MWNT surrounded by surfactant after sonication; (**c**) pyrrole adsorption on the MWNT surface; (**d**) polymerization of pyrrole on MWNT; (**e**) PPy coated MWNT after washing.

Figure 2 shows a schematic diagram of the polymerization procedure of pyrrole onto the carbon nanotubes in the presence of the surfactant. In accordance with an earlier study [22], SDBS molecules as a surfactant were absorbed firmly onto the MWNT surface, because of van der Waals intermolecular forces and has functionalized the MWNT to improve the dispersion of nanotubes inside the solution [22]. The Polypyrrole molecule could enter into this MWNT/SDBS solution and situate at the surface between surfactant and carbon nanotube [23–25].

### 3.2. Optical Measurements

**Figure 3:**
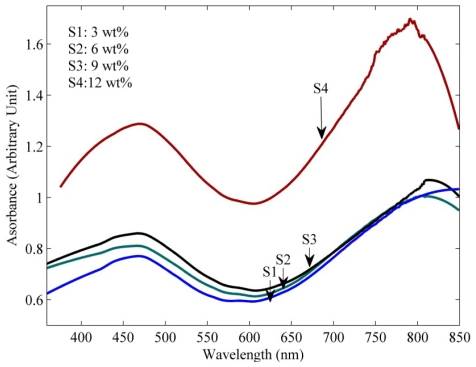
Absorbance spectrum of the PPy/MWNT for different MWNT (wt %) concentrations S1: 3 wt %; S2: 6 wt %; S3: 9 wt %; S4: 12 wt %.

The absorption spectra of the samples obtained from a UV-Vis spectrophotometer (Shimadzu-UV1650PC) are shown in Figure 3. The measurements of absorption spectra were carried out at room temperature for visible wavelength ranging from 350 nm to 850 nm. The linear absorption spectra values of the present samples were obtained to calculate the optical nonlinearities.

**Figure 4:**
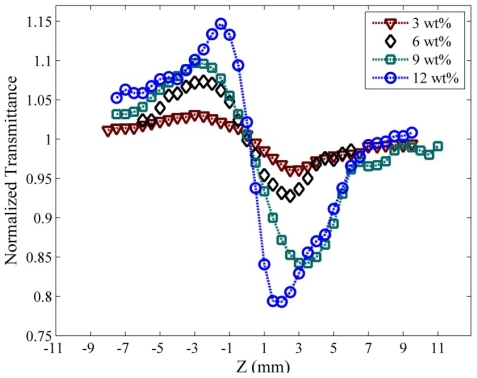
Normalized Z-scan transmittance curves of closed-aperture for PPy/MWNT for different MWNT (wt %) concentrations.

For optical nonlinearity measurements, a closed-aperture and an open-aperture have been used to estimate the nonlinear refraction coefficient and nonlinear absorption coefficient, respectively. The closed-aperture Z-scan curves for PPy/MWNT nanocomposite films are shown in Figure 4. The symbols represent the experimental data and the solid lines are theoretical fits to the closed-aperture and open-aperture Z-scan equations. The theoretical fits to the closed aperture (not shown) is used the standard equation given as [26].

(1) $$T(z,\Delta \varphi )=1-\frac{4\Delta \varphi_{o}x}{\left(x^{2}+1)(x^{2}+9\right)}$$

The nonlinear refractive index of the nanocomposite films was calculated using a simple relationship proposed by Sheik Bahaei *et al*. [26]:

(2) $$n_{2}=\Delta \varphi_{0}\lambda /2\pi I_{0}L_{eff}$$

where λ is the wavelength of the laser light, and I_0_ is the peak intensity within the sample. The terms Δφ_0_ and *L_eff_* are the nonlinear phase shift and the effective thickness, respectively, given by the following relationships [26,27]:

(3) $$\Delta \varphi_{0}=\frac{\Delta T_{p-v}}{0.406{\left(1-S\right)}^{0.25}}$$

(4) $$L_{eff}=[1-\text{exp}(-\alpha_{0}L)]/\alpha_{0}$$

Here, *L*, *S*, and *α*_0_ are sample thickness, aperture linear transmittance and linear absorption coefficient at wavelength λ, respectively. However, if the sample has a nonlinear refractive index and nonlinear absorption properties, the normalized transmission curve of closed-aperture data does not show a perfectly symmetrical curve. This phenomenon can be clearly seen in Figure 4.

**Figure 5:**
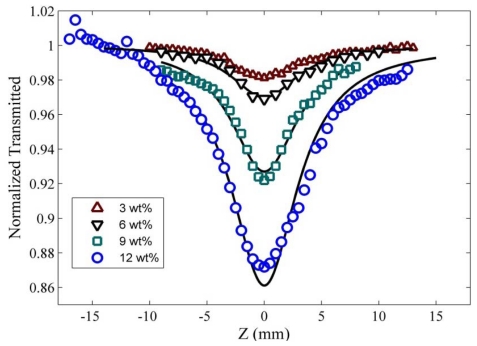
Normalized Z-scan transmittance curves of open-aperture for PPy/MWNT at different MWNT (wt %) concentrations.

Figure 5 shows the open aperture experimental data of samples obtained for carbon nanotubes at different concentrations using 532 nm excitation laser beam. The nonlinear absorption coefficient β (cm/W) can be determined from a best-fitting procedure performed on the experimental data of the open-aperture measurement using a well-known equation used by Sheik Bahae *et al*. [26,27]:

(5) $$T(z,s=1)=\sum_{m=0}^{\infty }\frac{{\left[-\beta I_{0}L_{eff}/(1+z/z_{0})\right]}^{m}}{{\left(m+1\right)}^{3/2}}$$

where *T*(*z*, *s* = 1) is the normalized transmittance for the open aperture (OA) with z_0_ being the Rayleigh range. In this work, we used the MATLAB software for fitting the experimental data with theoretical equations 1 and 5 for closed and open aperture measurements, respectively. The nonlinear refractive index n_2_ and nonlinear absorption coefficient β were used to calculate the real and imaginary parts of the third-order nonlinear optical susceptibility *χ*^3^ [28,29] according to the following relationships:

(6) $$\text{Re }\chi^{3}(esu)=10^{-4}\frac{{ɛ}_{0}{\text{c}}^{2}{\text{n}}_{0}^{2}}{\pi }n_{2}(cm^{2}W^{-1})$$

(7) $$\text{Im }\chi^{3}(esu)=10^{-2}\frac{{ɛ}_{0}{\text{c}}^{2}{\text{n}}_{0}^{2}\lambda }{4\pi^{2}}\beta (cmW^{-1})$$

where *ɛ*_0_ is the vacuum permittivity, and c is the velocity of light in a vacuum. The values of linear refractive index n_0_ were measured by using an ellipsometer (DRE-Dr.Riss Ellipsometerbau GmbH). Thus, the absolute value of the third-order nonlinear optical susceptibility was calculated as:

(8) $$|\chi^{3}|={\left[{\left(\text{Re}(\chi^{3})\right)}^{2}+{\left(\text{Im}(\chi^{3})\right)}^{2}\right]}^{1/2}$$

The values of the nonlinear refraction coefficient n_2_ (cm^2^/W), nonlinear absorption coefficient β (cm/W) and the third-order nonlinear susceptibility obtained for the present samples are listed in Table 1.

**Figure 6:**
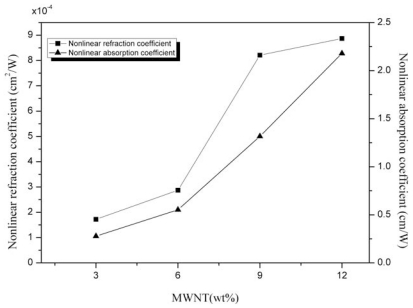
Variation of the nonlinear refraction coefficient and nonlinear absorption coefficient for different MWNT (wt %) concentrations.

The effects of concentration on nonlinear refraction and nonlinear absorption are shown in Figure 6. The nonlinear refractive index and the nonlinear absorption coefficient increased as the concentration of carbon nanotubes increased. With higher concentration of CNT more particles will interact with the laser beam and subsequently, more particles are thermally agitated resulting in enhanced effect on optical nonlinearities.

### 3.3. Conductivity Studies

**Figure 7:**
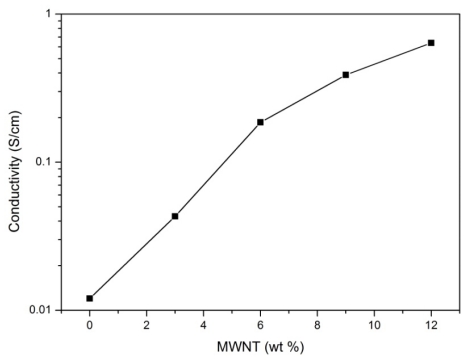
Variation of conductivity of the PPy/MWNT composite films for different MWNT concentration. The curve represents the best fit of the data to equation 1.

The variation of the conductivity of the thin film composite versus MWNT concentration is plotted in Figure 7. The results show that conductivity increases with increasing concentration of the MWNTs in the composite. This means the nanotubes improves the conductivity of the composite film by introducing a conductive network into the polymer matrix which improves the conductivity. This may be due to the larger surface area of MWNT that serve as a conducting bridge, connecting PPy conducting domains and increasing the effective percolation [30]. One of the interesting advantages obtained, is the possibility of modulating the charge transport properties of the composite film.

## 4. Conclusions

The presence of nanotube did not result in any significant degradation of absorption coefficient in the visible region. The nonlinear refractive index, n_2_, and nonlinear absorption coefficient, β, of PPy/MWNT were measured successfully for four different concentrations. The variation of the nonlinear coefficients of samples as concentrations increases was noted. In addition, third-order nonlinear optical susceptibilities were calculated using the measured values of n_2_ and β, and the results suggested a significant, third-order nonlinear response. The sign of the nonlinear refractive index was found to be negative and the magnitude was in the order of 10^−4^ cm^2^/W. It can be concluded that doping of PPy with MWNT increases the conductivity of the nanocomposite which is mainly due to the introduction of conducting paths in the polymer matrix.

## Figures and Tables

**Table 1:** The nonlinear optical parameters measured for PPy/MWNT at different concentrations.

Thin film samples	Concentration (wt %)	n_2_ (cm^2^/W) × 10^−4^	β (2PA) (cm/W)	Re (χ^(3)^) × 10^−4^	Im (χ^(3)^) × 10^−4^	|χ^(3)^| × 10^−4^
S1	3	−1.721	0.278	−85.461	0.5852	85.485
S2	6	−2.872	0.553	−142.612	1.1640	142.614
S3	9	−8.211	1.317	−407.951	2.769	407.959
S4	12	−8.872	2.179	−440.750	4.582	440.776
